# Research on the Line of Sight Stabilization Control Technology of Optronic Mast under High Oceanic Condition and Big Swaying Movement of Platform

**DOI:** 10.3390/s23063182

**Published:** 2023-03-16

**Authors:** Lintao Lan, Wei Jiang, Fangwu Hua

**Affiliations:** 1 State Key Laboratory of Digital Manufacturing Equipment and Technology, School of Mechanical Science and Engineering, Huazhong University of Science and Technology, Wuhan 430074, China; 2 Huazhong Institute of Electro-Optics, Wuhan National Laboratory for Optoelectronics, Wuhan 430223, China

**Keywords:** composite control method, optronic mast, line of sight, adaptive radial basis function neural network, sliding mode control, saturation function

## Abstract

To realize high-performance line of sight (LOS) stabilization control of the optronic mast under high oceanic conditions and big swaying movements of platforms, a composite control method based on an adaptive radial basis function neural network (RBFNN) and sliding mode control (SMC) is proposed. The adaptive RBFNN is used to approximate the nonlinear and parameter-varying ideal model of the optronic mast, so as to compensate for the uncertainties of the system and reduce the big-amplitude chattering phenomenon caused by excessive switching gain in SMC. The adaptive RBFNN is constructed and optimized online based on the state error information in the working process; therefore, no prior training data are required. At the same time, a saturation function is used to replace the sign function for the time-varying hydrodynamic disturbance torque and the friction disturbance torque, which further reduce the chattering phenomenon of the system. The asymptotic stability of the proposed control method has been proven by the Lyapunov stability theory. The applicability of the proposed control method is validated by a series of simulations and experiments.

## 1. Introduction

In recent years, the relatively popular underwater unmanned vehicle (UUV) has often been equipped with visible light cameras, infrared cameras, laser ranging modules, and so forth to obtain the water surface images and video information [1]. Under the high oceanic condition (the oceanic condition greater than or equal to level 4 for the UUV platform involved in this paper), the marine environmental loads such as waves and ocean currents act on the UUV directly [2], and cause the UUV to generate complex random swaying movements. This swaying movement is coupled to the imaging payloads through the installation base, which seriously affects the imaging quality. The optronic mast is a two-axis inertially stabilized platform (ISP) carrying imaging payloads, which can isolate the disordered disturbance by controlling the rotation of the pitch and azimuth axes, so that the imaging payloads can acquire clear and stable image information.

The optronic mast needs to pass through the sea level to acquire the sea surface image information, so it will be also directly affected by the disturbance torque of waves and ocean currents. In order to meet the pressure and sealing needs of the seawater, the azimuth gimbal adopts an oil-filled mechanical seal structure; this structure not only has large inertia, but also the friction resistance torque changes fiercely during startup and normal work. For the pitch axis, it is mainly subjected to the mass imbalance torque caused by a visible light camera, rate gyro, mechanical limit switch, and so forth. In addition, the time variation of the focal length of imaging payloads also affects the inertia tensor of the system. No IMU and other attitude sensors are installed on the optronic mast, and just the attitude angles of the base can be obtained from the platform, with no more disturbance information, such as the angular rate and angular acceleration of the platform. It can be seen that the optronic mast is a system of nonlinearity, parameter time variation, and strong disturbance. Under the high oceanic condition and big swaying movement of the platform (the swaying amplitude of platform is greater than 5 degrees for the studied optronic mast in this paper), how high performance for the stabilization control of the LOS can be achieved is a challenge.

The PID controller is commonly used in the LOS stable control of the optronic mast, and this control method has the advantages of simple structure, strong operability and wide application. In the Refs. [3,4,5], the fuzzy PID control method was developed to the stable control of ISP. Although this control method can adjust the PID control parameters adaptively, the control performance is easily affected by the external disturbance and system parameter variation. Bingul proposed an intelligent PID with the PD feedforward control method [6] and achieved a good simulation effect under the influence of ocean currents, external disturbances, and model parameter uncertainty, but it has not been tested in practice. The H∞ robust controller is insensitive to the external disturbance and system parameter variation, which can obtain an ideal control effect. Lee and Martin realized stable control by using the H∞ control method [7,8], but the control performance decreases with the increment of the system robustness. Furthermore, Ren carried out theoretical in-depth research on the finite-time $L_{2}$ control problem [9,10], where the sufficient conditions were given and proven for solving the finite-time $L_{2}$ asynchronous control gain. He designed the finite-time asynchronous controller and obtained good simulation results, but there is still a lot of work to be completed for engineering application. The LOS stable control of optronic mast is a chain control structure, where for this control structure, the back-stepping control can stabilize the LOS by linearizing the nonlinearity and step-by-step recursive process [11]. Thinh used a back-stepping control method to achieve the target tracking of a two-axis gimbal mechanism [12], and Setoodeh used a back-stepping control method to achieve the stable control of the shipboard camera [13], but the system parameters variation and disturbance may have a serious impact on the control performance [14]. Hamed established an uncertain linear ISP model, which highlights the influence of the cable restraint, stribeck friction, coulomb friction and other system disturbances [15]. In order to overcome the influence of system parameter variation and disturbances, some research has been performed on the model and estimation of the uncertainties of system. Li adopted a two-order Cascade Extended State Observe (CESO) and a reduced-order cascade extended state observer (RCESO), respectively to estimate the coupling torque, nonlinear friction and unmodeled dynamics, and explored a good angular speed tracking control method in double-gimbal control moment gyros [16,17]. Burak proposed a method to estimate the unknown disturbances of the system, and designed an integral linear quadratic regulator as the stabilizing controller in a two-axis gimbal system [18]. Meanwhile, a lot of research has been performed on the adaptive control (AC) to resolve the uncertainties of system. In the Refs. [19,20,21,22], these AC methods have dealt well with nonlinear disturbance and uncertainties of the system, and achieved good effects in simulations, but not been applied in the practical systems under input saturation constraints. Similar control methods have been proven effective in practical applications [23,24,25], but the control performance depends on the precise dynamic model of the system, which may lead to poor applicability under parameters variation of the system. The neural network has good approximation ability to the disturbance of the nonlinear system with parameters variation [26,27]. However, as neural network control needs a lot of time and sample training data to optimize the neural network, it is difficult to obtain comprehensive and sufficient sample data.

Under the high oceanic condition and the large swaying movement of platforms, to achieve high-performance LOS stabilization control with a fast dynamic response and highly stable precision of the optronic mast, motivated by the above methods, a composite control method is proposed based on adaptive RBFNN and SMC in this paper. Based on the system state error, the adaptive RBFNN was constructed and optimized online, and no prior training data were required. The adaptive RBFNN is used to approximate the ideal model of the nonlinear and time variation system, so as to compensate for the uncertainties and coupling disturbance of the system, and avoid excessive switching gain in SMC. For the time-varying hydrodynamic disturbance torque and friction disturbance torque, a saturation function was used to further reduce the system’s chattering problem instead of the sign function. The asymptotic stability of the proposed control method was proved by using the Lyapunov stability theory.

The outline of this paper is organized as follows: in Section 2, the nonlinear dynamic model of the optronic mast is constructed. In Section 3, the composite control method proposed is introduced, and the approximation law online and the control law is established. A series of simulations and experiments validate the effectiveness of the proposed control method in Section 4, followed by the conclusion in Section 5.

## 2. The Nonlinear Dynamic Model of the Optronic Mast

**Figure 1:**
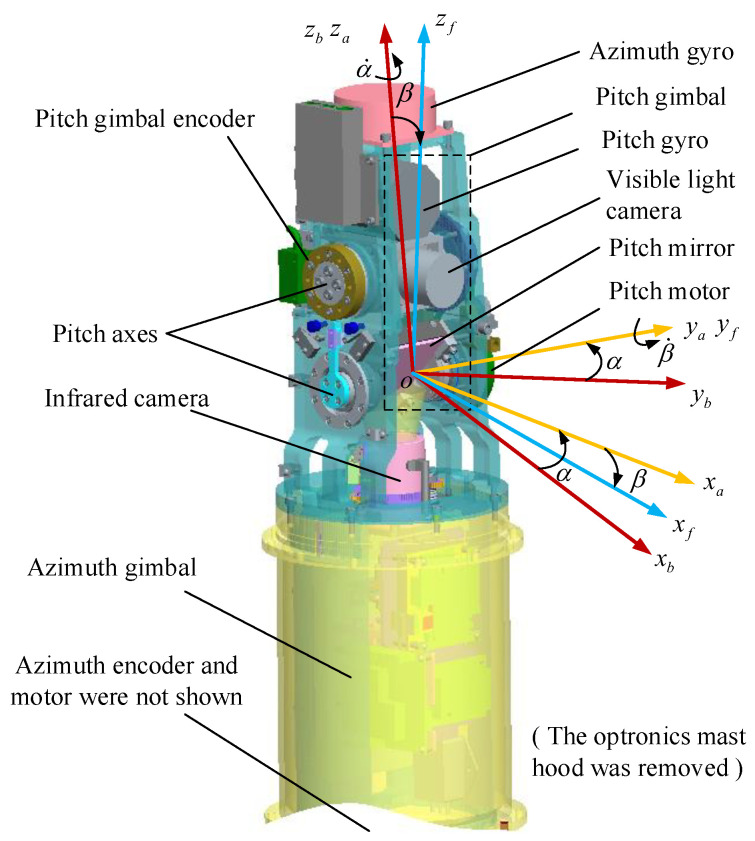
The 3D model of optronic mast and corresponding coordinate system.

As shown in Figure 1, the optronic mast is a two-axis ISP, which consists of an outer azimuth gimbal and an inner pitch gimbal. The infrared camera mirror, visible light camera, mechanical limit switch, and so forth are mounted on the pitch gimbal. Two fiber optic gyroscopes are mounted on two gimbals respectively to provide the corresponding angular velocity information. Then two encoders are mounted on two gimbals respectively to provide the relative rotation angular displacement of two gimbals. The optronic mast is installed on the base of the UUV, and the base inertial attitude information is provided by the inertial measurement unit of the UUV. Based on the measured information of gyros, encoders and inertial measurement unit, the servo-controller generates corresponding control signals to adjust the LOS of imaging sensors to obtain precise images and videos of the sea surface target.

Three Cartesian coordinate systems are established, as shown in Figure 1. The base coordinate system $o-x_{b}y_{b}z_{b}$ is fixed to the installation base, the azimuth coordinate system $o-x_{a}y_{a}z_{a}$ is fixed to the azimuth axis, and the pitch coordinate system $o-x_{f}y_{f}z_{f}$ is fixed to the pitch axis, $x_{b},y_{b},z_{b},x_{a},y_{a},z_{a}$ and $x_{f},y_{f},z_{f}$ are the corresponding coordinate axes of them. The azimuth coordinate system has only one azimuth angle α rotating around the axis $oz_{a}$ relative to the base coordinate system, and the pitch coordinate system has only one pitch angle β rotating around the axis $oy_{f}$ relative to the azimuth coordinate system, the two angle values are measured by the encoders on the gimbals. $T_{b}^{a}$ is the transformation matrix from the base coordinate system to azimuth coordinate system, and $T_{a}^{f}$ is the transformation matrix from the azimuth coordinate system to pitch coordinate system.

(1) $$T_{b}^{a}=\left[\begin{array}{ccc} \cos\alpha & \sin\alpha & 0\\ -\sin\alpha & \cos\alpha & 0\\ 0 & 0 & 1 \end{array}\right]$$

(2) $$T_{a}^{f}=\left[\begin{array}{ccc} \cos\beta & 0 & -\sin\beta \\ 0 & 1 & 0\\ \sin\beta & 0 & \cos\beta \end{array}\right]$$

In inertial space, the attitude angle and angular rate of the installation base are coupled to the azimuth axis and the pitch axis by the structural joint. Let $\theta_{ib}^{b}$, $\theta_{ia}^{a}$ and $\theta_{if}^{f}$ represent the inertial space attitude angles of the installation base expressed in the base coordinate, the azimuth axis expressed in the azimuth coordinate, the pitch axis expressed in the pitch coordinate, respectively; similarly, let $\omega_{ib}^{b}$, $\omega_{ia}^{a}$ and $\omega_{if}^{f}$ represent the inertial space angular rates of them. Based on the coordinate transformation matrixes, the attitude angles and angular rates of azimuth and pitch axes can be obtained as

(3) $$\theta_{ia}^{a}=T_{b}^{a}\cdot \theta_{ib}^{b}+\theta_{ba}^{a}=\left[\begin{array}{c} \psi \cos\alpha +\varphi \sin\alpha \\ -\psi \sin\alpha +\varphi \cos\alpha \\ \zeta +\alpha \end{array}\right]$$

(4) $$\theta_{if}^{f}=T_{a}^{f}\cdot \theta_{ia}^{a}+\theta_{af}^{f}=\left[\begin{array}{c} \theta_{iax}^{a}\cos\beta -\theta_{iaz}^{a}\sin\beta \\ \theta_{iay}^{a}+\beta \\ \theta_{iax}^{a}\sin\beta +\theta_{iaz}^{a}\cos\beta \end{array}\right]$$

(5) $$\omega_{ia}^{a}=T_{b}^{a}\cdot \omega_{ib}^{b}+\omega_{ba}^{a}=\left[\begin{array}{c} \omega_{ibx}^{b}\cos\alpha +\omega_{iby}^{b}\sin\alpha \\ -\omega_{ibx}^{b}\sin\alpha +\omega_{iby}^{b}\cos\alpha \\ \omega_{ibz}^{b}+\alpha \end{array}\right]$$

(6) $$\omega_{if}^{f}=T_{a}^{f}\cdot \omega_{ia}^{a}+\omega_{af}^{f}=\left[\begin{array}{c} \omega_{iax}^{a}\cos\beta -\omega_{iaz}^{a}\sin\beta \\ \omega_{iay}^{a}+\beta \\ \omega_{iax}^{a}\sin\beta +\omega_{iaz}^{a}\cos\beta \end{array}\right]$$

where $\theta_{ba}^{a}={\left[\begin{array}{ccc} 0 & 0 & \alpha \end{array}\right]}^{T}$ and $\theta_{af}^{f}={\left[\begin{array}{ccc} 0 & \beta & 0 \end{array}\right]}^{T}$ are the relative rotation angles measured by the corresponding encoder, $\omega_{ba}^{a}={\left[\begin{array}{ccc} 0 & 0 & \dot{\alpha } \end{array}\right]}^{T}$ and $\omega_{af}^{f}={\left[\begin{array}{ccc} 0 & \dot{\beta } & 0 \end{array}\right]}^{T}$ are the corresponding relative angular rates. $\theta_{ib}^{b}={\left[\begin{array}{ccc} \psi & \phi & \zeta \end{array}\right]}^{T}$ can be measured and provided by the inertial measurement unit of UUV, $\omega_{iaz}^{a}$ and $\omega_{ify}^{f}$ can be measured by the corresponding fiber optical gyro, the subscripts x,y and *z* denote the components expressed in the corresponding coordinate axis of the coordinate systems. ${\left[\begin{array}{cccc} \theta_{ify}^{f} & \omega_{ify}^{f} & \theta_{iaz}^{a} & \omega_{iaz}^{a} \end{array}\right]}^{T}$ is chosen as the criteria and state variables of the LOS of optronic mast.

By the Newton–Euler theory, the dynamic model of the pitch gimbal can be obtained as the following [28]:

(7) $$M_{f}=J_{f}{\dot{\omega }}_{if}^{f}+\omega_{if}^{f}\times \left(J_{f}\omega_{if}^{f}\right)$$

$M_{f}={\left[\begin{array}{ccc} M_{fx} & M_{fy} & M_{fz} \end{array}\right]}^{T}$ denotes the inertia torque of the pitch gimbal, and the $J_{f}$ denotes the inertia matrix of the pitch gimbal. Assuming that the inertia principal axis of the pitch gimbal is the pitch axis, that is, the inertia products of $J_{f}$ are all zeros, $J_{f}=\mathrm{diag}\left(J_{fxx},J_{fyy},J_{fzz}\right)$. Therefore, the component on the coordinate axis $y_{f}$ of Equation (7) can be written as:

(8) $$M_{fy}=J_{fyy}{\dot{\omega }}_{ify}^{f}+\left(J_{fxx}-J_{fzz}\right)\omega_{ifx}^{f}\omega_{ifz}^{f}$$

Similarly, the dynamic model of the azimuth gimbal can be obtained as the following:

(9) $$M_{a}=\frac{d}{dt}\left(J_{a}^{z}\omega_{ia}^{a}\right)+\omega_{ia}^{a}\times \left(J_{a}^{z}\omega_{ia}^{a}\right)$$

$M_{a}={\left[\begin{array}{ccc} M_{ax} & M_{ay} & M_{az} \end{array}\right]}^{T}$ denotes the inertia torque of the azimuth gimbal. Considering the coupling effect of the inertia moment of the pitch gimbal on the azimuth gimbal, the inertia moment of the azimuth gimbal should be calculated as the following:

(10) $$J_{a}^{z}=J_{a}+T_{f}^{a}J_{f}{\left(T_{f}^{a}\right)}^{T}$$

where $J_{a}$ denotes the inertia matrix of the azimuth gimbal excluding the pitch gimbal, similar to the pitch gimbal, and the $J_{a}$ can be expressed as $J_{a}=\mathrm{diag}\left(J_{axx},J_{ayy},J_{azz}\right)$. Substituting the Equation (10) into the Equation (9), the dynamic model of the azimuth gimbal is given by

(11) $$\begin{array}{c} M_{az}=J_{azz}{\dot{\omega }}_{iaz}^{a}-J_{fxx}{\dot{\omega }}_{ifx}^{f}\sin\beta -J_{fxx}\omega_{ifx}^{f}\dot{\beta }\cos\beta +J_{fzz}{\dot{\omega }}_{ifz}^{f}\cos\beta -J_{fzz}\omega_{ifz}^{f}\dot{\beta }\sin\beta \;\\ \;\;\;\;\;\;\;\;\;+\omega_{iax}^{a}\left[J_{ayy}\omega_{iay}^{a}+J_{fyy}\omega_{ify}^{f}\right]-\omega_{iay}^{a}\left[J_{axx}\omega_{iax}^{a}+J_{fxx}\omega_{ifx}^{f}\cos\beta +J_{fzz}\omega_{ifz}^{f}\sin\beta \right] \end{array}$$

Both the pitch axis and azimuth axis are driven directly by the DC torque motors, and the dynamic model of the motor mounted on the pitch axis is given by

(12) $$\left\{\begin{array}{c} T_{mf}=K_{tf}i_{mf}\;\;\;\;\;\;\;\;\;\;\;\;\;\;\;\;\;\;\;\;\;\\ e_{ef}=K_{ef}\omega_{amf}\;\;\;\;\;\;\;\;\;\;\;\;\;\;\;\;\;\;\;\\ u_{f}=e_{ef}+R_{mf}i_{mf}+L_{mf}\frac{di_{mf}}{dt} \end{array}\right.$$

where $T_{mf}$ is the motor total torque, $K_{tf}$ is the torque sensitivity, $i_{mf}$ is the current of the motor armature, $e_{ef}$ is the back electromotive force(EMF), $K_{ef}$ is the back-EMF coefficient [29], $\omega_{amf}$ is the rotational angular velocity of the motor relative to the azimuth gimbal, and $\omega_{amf}=\omega_{ify}^{f}-\omega_{iay}^{f}$, $u_{f}$ is the input voltage of the motor, $R_{mf}$ is the armature resistance of the motor, $L_{mf}$ is the armature inductance of the motor, and the subscript *f* denotes the pitch motor. Since the value of $L_{mf}$ is often very small, it can be ignored, and the $T_{mf}$ is expressed as

(13) $$T_{mf}=K_{tf}\frac{u_{f}-K_{ef}\left(\omega_{ify}^{f}-\omega_{iay}^{f}\right)}{R_{mf}}$$

The dynamics of the pitch axis is given by

(14) $$\left\{\begin{array}{c} M_{fy}=M_{o}-T_{df}\;\;\;\;\;\;\;\;\;\;\;\;\;\;\\ J_{mf}{\dot{\omega }}_{imf}=T_{mf}-T_{dmf}-M_{o} \end{array}\right.$$

where $M_{o}$ is the output torque of the pitch motor, which is used to drive the pitch axis, $T_{df}$ is the friction torque of the pitch axis, $J_{mf}$ and $T_{dmf}$ are the inertia moment and the friction torque of the pitch motor armature, respectively, ${\dot{\omega }}_{imf}$ is the angular acceleration of the pitch motor, and ${\dot{\omega }}_{imf}={\dot{\omega }}_{ify}^{f}$. Substituting the Equation (13) into the Equation (14) to obtain

(15) $$M_{fy}=K_{tf}\frac{u_{f}-K_{ef}\left(\omega_{ify}^{f}-\omega_{iay}^{f}\right)}{R_{mf}}-T_{dmf}-T_{df}-J_{mf}{\dot{\omega }}_{ify}^{f}$$

Similarly, the torque acting on the azimuth axis is expressed as

(16) $$M_{az}=K_{ta}\frac{u_{a}-K_{ea}\left(\omega_{iaz}^{a}-\omega_{ibz}^{a}\right)}{R_{ma}}-T_{dma}-T_{da}-T_{w}-J_{ma}{\dot{\omega }}_{iaz}^{a}$$

The difference is that the azimuth axis is also subjected to the hydrodynamic disturbance torque $T_{w}$, while the pitch axis is protected from the $T_{w}$ by the hood. Substituting the Equations (15) and (16) into the Equations (8) and (11), respectively, the dynamic models of the optronic mast are expressed as

(17) $$\begin{array}{c} {\dot{\omega }}_{ify}^{f}=-\frac{K_{tf}K_{ef}}{\left(J_{fyy}+J_{mf}\right)R_{mf}}\omega_{ify}^{f}+\frac{\left(J_{fzz}-J_{fxx}\right)\omega_{iax}^{a}\cos\left(2\beta \right)}{J_{fyy}+J_{mf}}\omega_{iaz}^{a}+\;\\ \;\;\;\;\;\;\;\;\;\;\;\frac{\left(J_{fzz}-J_{fxx}\right)\left(\omega {{}_{iax}^{a}}^{2}-\omega {{}_{iaz}^{a}}^{2}\right)\sin\left(2\beta \right)}{2(J_{fyy}+J_{mf})}+\frac{K_{tf}K_{ef}\omega_{iay}^{a}}{\left(J_{fyy}+J_{mf}\right)R_{mf}}+\frac{K_{tf}}{\left(J_{fyy}+J_{mf}\right)R_{mf}}u_{f}-\frac{T_{dmf}+T_{df}}{J_{fyy}+J_{mf}} \end{array}$$

(18) $$\begin{array}{c} {\dot{\omega }}_{iaz}^{a}=\frac{\left(J_{fzz}-J_{fxx}\right)\left(2\dot{\beta }+\omega_{iay}^{a}\right)\sin\left(2\beta \right)R_{ma}-2K_{ta}K_{ea}}{2\left(J_{azz}+J_{fzz}\cos^{2}\beta +J_{fxx}\sin^{2}\beta +J_{ma}\right)R_{ma}}\omega_{iaz}^{a}\;\\ \;\;\;\;\;\;\;\;-\frac{J_{fyy}\omega_{iax}^{a}}{J_{azz}+J_{fzz}\cos^{2}\beta +J_{fxx}\sin^{2}\beta +J_{ma}}\omega_{ify}^{f}\;\\ \;\;\;\;\;\;\;\;-\frac{\left(J_{ayy}-J_{axx}-J_{fxx}\cos^{2}\beta -J_{fzz}\sin^{2}\beta \right)\omega_{iay}^{a}+\left(J_{fzz}-J_{fxx}\right)\dot{\beta }\cos\left(2\beta \right)}{J_{azz}+J_{fzz}\cos^{2}\beta +J_{fxx}\sin^{2}\beta +J_{ma}}\omega_{iax}^{a}\;\\ \;\;\;\;\;\;\;\;-\frac{\left(J_{fzz}-J_{fxx}\right)\sin\left(2\beta \right)}{2(J_{azz}+J_{fzz}\cos^{2}\beta +J_{fxx}\sin^{2}\beta +J_{ma})}{\dot{\omega }}_{iax}^{a}\;\\ \;\;\;\;\;\;\;\;+\frac{K_{ta}K_{ea}}{\left(J_{azz}+J_{fzz}\cos^{2}\beta +J_{fxx}\sin^{2}\beta +J_{ma}\right)R_{ma}}\omega_{ibz}^{b}\;\\ \;\;\;\;\;\;\;\;+\frac{K_{ta}}{\left(J_{azz}+J_{fzz}\cos^{2}\beta +J_{fxx}\sin^{2}\beta +J_{ma}\right)R_{ma}}u_{a}-\frac{T_{dma}+T_{da}+T_{w}}{J_{azz}+J_{fzz}\cos^{2}\beta +J_{fxx}\sin^{2}\beta +J_{ma}} \end{array}$$

For simplicity, it can be defined as follows:

(19) $$\begin{array}{c} F_{1}=-\frac{K_{tf}K_{ef}}{\left(J_{fyy}+J_{mf}\right)R_{mf}}\omega_{ify}^{f}+\frac{\left(J_{fzz}-J_{fxx}\right)\omega_{iax}^{a}\cos\left(2\beta \right)}{J_{fyy}+J_{mf}}\omega_{iaz}^{a}\;\\ \;\;\;\;\;\;\;+\frac{\left(J_{fzz}-J_{fxx}\right)\left(\omega {{}_{iax}^{a}}^{2}-\omega {{}_{iaz}^{a}}^{2}\right)\sin\left(2\beta \right)}{2(J_{fyy}+J_{mf})}+\frac{K_{tf}K_{ef}\omega_{iay}^{a}}{\left(J_{fyy}+J_{mf}\right)R_{mf}} \end{array}$$

(20) $$B_{1}=\frac{K_{tf}}{\left(J_{fyy}+J_{mf}\right)R_{mf}}$$

(21) $$D_{1}=\frac{-T_{dmf}-T_{df}}{J_{fyy}+J_{mf}}$$

(22) $$\begin{array}{c} F_{2}=\frac{\left(J_{fzz}-J_{fxx}\right)\left(2\dot{\beta }+\omega_{iay}^{a}\right)\sin\left(2\beta \right)R_{ma}-2K_{ta}K_{ea}}{2\left(J_{azz}+J_{fzz}\cos^{2}\beta +J_{fxx}\sin^{2}\beta +J_{ma}\right)R_{ma}}\omega_{iaz}^{a}\;\\ \;\;\;\;\;\;\;\;\;-\frac{J_{fyy}\omega_{iax}^{a}}{J_{azz}+J_{fzz}\cos^{2}\beta +J_{fxx}\sin^{2}\beta +J_{ma}}\omega_{ify}^{f}\;\\ \;\;\;\;\;\;\;\;\;-\frac{\left(J_{ayy}-J_{axx}-J_{fxx}\cos^{2}\beta -J_{fzz}\sin^{2}\beta \right)\omega_{iay}^{a}+\left(J_{fzz}-J_{fxx}\right)\dot{\beta }\cos\left(2\beta \right)}{J_{azz}+J_{fzz}\cos^{2}\beta +J_{fxx}\sin^{2}\beta +J_{ma}}\omega_{iax}^{a}\;\\ \;\;\;\;\;\;\;\;\;-\frac{\left(J_{fzz}-J_{fxx}\right)\sin\left(2\beta \right)}{2(J_{azz}+J_{fzz}\cos^{2}\beta +J_{fxx}\sin^{2}\beta +J_{ma})}{\dot{\omega }}_{iax}^{a}\;\\ \;\;\;\;\;\;\;\;\;+\frac{K_{ta}K_{ea}}{\left(J_{azz}+J_{fzz}\cos^{2}\beta +J_{fxx}\sin^{2}\beta +J_{ma}\right)R_{ma}}\omega_{ibz}^{b} \end{array}$$

(23) $$B_{2}=\frac{K_{ta}}{\left(J_{azz}+J_{fzz}\cos^{2}\beta +J_{fxx}\sin^{2}\beta +J_{ma}\right)R_{ma}}$$

(24) $$D_{2}=\frac{-T_{dma}-T_{da}-T_{w}}{J_{azz}+J_{fzz}\cos^{2}\beta +J_{fxx}\sin^{2}\beta +J_{ma}}$$

Then, the Equations (17) and (18) can be simplified as:

(25) $${\dot{\omega }}_{ify}^{f}=F_{1}+B_{1}u_{f}+D_{1}$$

(26) $${\dot{\omega }}_{iaz}^{a}=F_{2}+B_{2}u_{a}+D_{2}$$

## 3. The Control System of the Optronic Mast

In the practical physical model of the optronic mast, the pitch axis and azimuth axis are not the inertia principal axes, the inertia products in the inertia matrixes $J_{f}$ and $J_{a}$ of the pitch and azimuth gimbals are not zeros, which makes the system characteristic $F_{i}\left(i=1,2\right)$ have an unknown component $\Delta F_{i}\left(i=1,2\right)$, and the disturbance $D_{i}\left(i=1,2\right)$ have an unknown component $\Delta D_{i}\left(i=1,2\right)$ [30]. Meanwhile, the time variation of the focal length of the imaging payloads will also cause the change of the system inertia moment. $F_{i}+\Delta F_{i}+\Delta D_{i}\left(i=1,2\right)$ represents the nonlinear and parameters-varying characteristic of the optronic mast and cannot be obtained exactly, thus they need to be estimated by some system parameters.

Let $x_{1}=\theta_{ify}^{f}$, $x_{2}=\omega_{ify}^{f}$, $x_{3}=\theta_{iaz}^{a}$ and $x_{4}=\omega_{iaz}^{a}$, then the state-space equation of the optronic mast is expressed as

(27) $$\left\{\begin{array}{l} {\dot{x}}_{1}=x_{2}\\ {\dot{x}}_{2}=F_{1}+\Delta F_{1}+B_{1}u_{f}+D_{1}+\Delta D_{1}\\ {\dot{x}}_{3}=x_{4}\\ {\dot{x}}_{4}=F_{2}+\Delta F_{2}+B_{2}u_{a}+D_{2}+\Delta D_{2} \end{array}\right.$$

**Figure 2:**
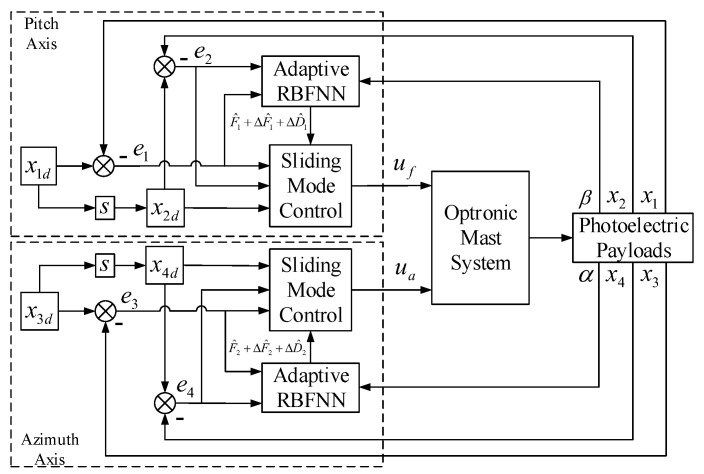
The block diagram of the proposed control method.

For the optronic mast system described in Equation (27), under the high oceanic condition and big swaying movement of UUV, the nonlinear characteristic $F_{i}+\Delta F_{i}\left(i=1,2\right)$ and the disturbance $D_{i}+\Delta D_{i}$ of an optronic mast will change dramatically with time. In this case, the use of the general feedback control method makes it difficult to achieve system stability. For example, with the commonly used PID control method, in order to make the system resist this strong disturbance, the general approach is to increase the PID control parameters, especially to increase the proportional parameter. According to experience, the increase in PID controller parameters will easily lead to control failure. At this time, the SMC controller naturally becomes a better choice, because it is almost unaffected by system parameters variation and external disturbance, and once the input meets some conditions, the system will be stabilized. However, in the absence of an accurate physical model of the system, the SMC needs to increase the switching gain to resist the influence of nonlinearity and parameters variation of the system, and pull the system from any state back to the specified sliding mode surface for stabilization control, which will cause a large amplitude chattering phenomenon. With regard to how to reduce the switching gain, a natural idea is to estimate the uncertainty caused by the nonlinearity and parameters variation of the system online, and effectively compensate for the switching gain in time. Therefore, an adaptive RBFNN can be used to approximate the ideal model of the system by the state errors in the working process, which can effectively reduce the switching gain and chattering amplitude. For the time-varying hydrodynamic disturbance torque and friction disturbance torque, a saturation function can be used to replace the sign switching function, which will further reduce the chattering phenomenon of the system. Based on the above ideas, this paper proposes a composite control method based on adaptive RBFNN and SMC. the block diagram of the proposed control algorithm is shown in Figure 2:

The $x_{1d}$ and $x_{3d}$ are target attitude angles of the LOS, and the $x_{2d}$ and $x_{4d}$ are intermediate target angular velocities of the LOS. The aim of the control is to obtain the control laws $u_{f}$ and $u_{a}$ by the proposed control method, so that the attitude angles $x_{1}$ and $x_{3}$ can accurately track the target attitude angles $x_{1d}$ and $x_{3d}$. The control processes of pitch axis and azimuth axis are basically the same, where for the pitch axis, the design of the proposed control method is described as follows.

Step 1: The sliding mode control law design.

Define the state error as

(28) $$e_{1}=x_{1d}-x_{1}$$

(29) $$e_{2}=x_{2d}-x_{2}$$

Define the sliding surface as [31]

(30) $$s={\dot{e}}_{1}+K_{1}e_{1}$$

where s=0 is the sliding surface, and $K_{1}>0$ is the parameter which decides the bandwidth of the state error.

Differentiate the Equation (30) with respect to time, and using

(31) $$\dot{s}={\dot{x}}_{2d}-F_{1}-\Delta F_{1}-B_{1}u_{f}-D_{1}-\Delta D_{1}+K_{1}e_{2}$$

Then the control law $u_{f}$ can be designed as

(32) $$u_{f}=\frac{{\dot{x}}_{2d}-F_{1}+K_{1}e_{2}+\eta \mathrm{sgn}\left(s\right)}{B_{1}}\;$$

where η is the upper bound of $D_{1}+\Delta D_{1}+\Delta F_{1}$. The first Lyapunov function is chosen as

(33) $$V_{1}=\frac{1}{2}s^{2}$$

Differentiate $V_{1}$ with respect to time, and then

(34) $$\begin{array}{c} {\dot{V}}_{1}=s\cdot \dot{s}\;\\ \;\;\;\;\;=s(-\eta \mathrm{sgn}\left(s\right)-D_{1}-\Delta D_{1}-\Delta F_{1})\;\\ \;\;\;\;\;=-\eta \left|s\right|-s\cdot \left(D_{1}+\Delta D_{1}+\Delta F_{1}\right)\leq 0 \end{array}$$

According to the LaSalle Invariance Principle, the closed-loop system is asymptotically stable, and the state error $e_{1}$ approaches zero at an exponential rate. However, with the increment of the system parameter variation and disturbance, and the nonlinear term $F_{1}$ being unable to be obtained exactly, then the η will be a large value, and it will cause a severe chattering problem due to excessive switching gain.

Step 2: The adaptive RBFNN approximation law design.

**Figure 3:**
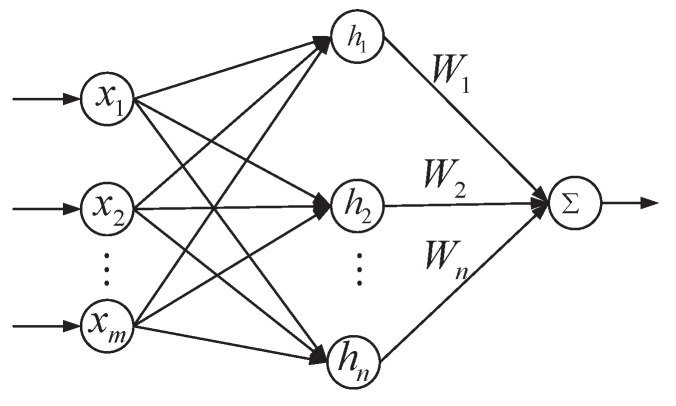
The schematic diagram of the RBFNN.

As shown in Figure 3, the RBFNN is a three-layer forward neural network, which consists of an input layer, hidden layer and output layer. The input layer consists of *m* input elements $x_{i}$, which only plays the role of data input, and the connection weight between the input layer and the hidden layer is 1. The hidden layer has *n* radial base neurons $h_{i}$, which make a local response to the input. When the input is near the central range of the basis function, the hidden node will produce a large output, and when it is far away from the central point, the output will decay exponentially. $W_{i}$ is the weight coefficient of the output, as many as the hidden nodes, and the final output is the linearly weighted x of the output of the neurons in the hidden layer.

With its universal approximation capabilities, the RBFNN with a limited number of hidden neurons can approximate a nonlinear function with any desired accuracy online. Therefore, the RBFNN can be used to estimate the nonlinear term and disturbance term of the system, then $F_{1}+\Delta F_{1}+\Delta D_{1}$ is expressed as

(35) $$F_{1}+\Delta F_{1}+\Delta D_{1}={W^{*}}^{T}h\left(x\right)+\varepsilon$$

where $x\in R^{1\times l}$ is the input of RBFNN, $h\left(x\right)\in R^{n\times 1}$ is the radial basis function of the hidden layer, ${W^{*}}^{T}\in R^{1\times n}$ is the weight matrix, and ε∈R is the bounded approximation error. In this paper, the Gaussian function is selected as the radial basis function:

(36) $$h_{i}=\exp\left(-\frac{{∥x-c_{i}∥}^{2}}{2{b_{i}}^{2}}\right)\;\;\left(i=1,2,\cdots ,n\right)$$

where $c_{i}$ and $b_{i}$ are the center and width of the Gaussian function, and *i* is the number of the hidden layers.

Therefore, the control law of the pitch axis can be rewritten as

(37) $$u_{f}=\frac{{\dot{x}}_{2d}-{\hat{W}}^{T}h\left(x\right)+K_{1}e_{2}+\lambda \mathrm{sgn}\left(s\right)}{B_{1}},\left(\lambda >D_{1}+\varepsilon \right)$$

where $\hat{W}$ is the estimated weight matrix. Define the error vector of the ideal weight matrix and the estimated weight matrix:

(38) $$\tilde{W}=W^{*}-\hat{W}$$

In order to obtain the approximation law, the second Lyapunov function is selected as:

(39) $$V_{2}=\frac{1}{2}s^{2}+\frac{1}{2}K_{2}{\tilde{W}}^{T}\tilde{W}\;\left(K_{2}>0\right)$$

Obviously, $V_{2}$ is positive definite. Differentiate $V_{2}$ with respect to time, and using

(40) $$\begin{array}{c} \begin{array}{c} {\dot{V}}_{2}=s\cdot \dot{s}+K_{2}{\tilde{W}}^{T}\dot{\tilde{W}}\;\\ \;\;\;\;\;=s\cdot \left(-F_{1}-\Delta F_{1}-\Delta D_{1}+{\hat{W}}^{T}h\left(x\right)-\lambda \mathrm{sgn}\left(s\right)-D_{1}\right)-K_{2}{\tilde{W}}^{T}\dot{\hat{W}}\;\\ \;\;\;\;\;=s\cdot \left(-{W^{*}}^{T}h\left(x\right)-\varepsilon +{\hat{W}}^{T}h\left(x\right)-\lambda \mathrm{sgn}\left(s\right)-D_{1}\right)-K_{2}{\tilde{W}}^{T}\dot{\hat{W}}\;\\ \;\;\;\;\;=-{\tilde{W}}^{T}\left(s\cdot h\left(x\right)+K_{2}\dot{\hat{W}}\right)-\lambda \left|s\right|-s\left(\varepsilon +D_{1}\right) \end{array} \end{array}$$

Therefore, the approximation law $\dot{\hat{W}}$ can be defined as

(41) $$\dot{\hat{W}}=-\frac{s\cdot h(x)}{K_{2}}$$

where $K_{2}$ is the learning rate, and then

(42) $${\dot{V}}_{2}=-\lambda \left|s\right|-s\left(\varepsilon +D_{1}\right)\leq 0$$

Therefore, the closed-loop system will be asymptotically stable by the LaSalle invariance principle.

In order to further reduce the chattering phenomenon caused by the switching action of λsgn(s), the saturation function sat(s) is used to replace the sign function sgn(s) in the control law Equation (37) [32], and the sat(s) is expressed as

(43) $$\mathrm{sat}\left(s\right)=\left\{\begin{array}{c} 1\;\;\;\;\;\;\;\;\;\;s>\Delta \;\;\;\;\;\;\;\;\;\;\;\;\;\;\;\\ k_{3}s\;\;\;\;\;\;\;\;\;\left|s\right|<\Delta ,k_{3}=1/\Delta \\ -1\;\;\;\;\;\;\;\;s<-\Delta \;\;\;\;\;\;\;\;\;\;\;\;\;\; \end{array}\right.$$

where Δ is the selected boundary layer.

With the control law designed by Equation (37) and the approximation law designed by Equation (41), the sliding mode surface s=0 is reachable, and the attitude angle of the pitch axis is globally convergent. Therefore, the attitude angle $x_{1}$ can track the target attitude angle $x_{1d}$ effectively.

## 4. Simulations and Experiments

### 4.1. Hardware System

The optronic mast is a slender cylinder, 1300 mm in height, maximum 160 mm in diameter, and its total weight is about 41 kg. Two fiber optic gyroscopes mounted on the pitch and azimuth gimbals, respectively were used to measure the inertia angular velocity information, whose zero deviation stability is $0.15^{\circ }/h$, and the data update period is 2 ms. A circular grating generated by RENISHAW was mounted on the pitch gimbal, and used to measure the angular displacement relative to the azimuth gimbal, whose precision is about 0.291 mrad. A resolver is mounted on the azimuth gimbal and is used to measure the angular displacement relative to the base, whose precision is about 14.5 μrad. No IMU or other attitude sensor is mounted on the optronic mast, and the LOS stable control is realized by the measured information of the above sensors and the base attitude provided by UUV. In addition, the pitch axis and the azimuth axis are driven directly by two DC torque motors, and the electrical parameters of the two motors are shown in Table 1.

Furthermore, the inertia moments $J_{f}$ and $J_{a}$ of two gimbals are estimated by system parameters, $J_{f}=\left[\begin{array}{ccc} 3.15 & 1.94 & 2.35 \end{array}\right]\times 10^{-3}$ kgm^2^ and $J_{a}=\left[\begin{array}{ccc} 23.6 & 23.6 & 0.22 \end{array}\right]$ kgm^2^. The friction torque of the pitch axis and azimuth axis is mainly generated by the preload of angular contact bearings. After the angular contact bearings are preloaded, $T_{zf}$ and $T_{za}$ are measured as 0.02 Nm, 0.5 Nm, respectively. In addition, the payloads mounted on the pitch gimbal produce significant mass imbalance torque, and the maximum value of $T_{mf}$ is measured as 0.1 Nm. An oil-filled and water-blocking mechanical seal device is mounted on the azimuth gimbal, whose maximum static friction torque is $T_{ja}=6.2$ Nm when starting. For the two DC motors, the maximum static friction torque of the armatures are measured as $T_{dmf}=0.008\;\mathrm{Nm}$, $T_{dma}=0.7\;\mathrm{Nm}$, respectively.

### 4.2. Simulations

Since the stable control performance of the LOS is seriously influenced by the non-ideal attitude perturbations of the UUV generated by ocean environmental loads such as waves and currents, friction torque and mass imbalance torque, a series of disturbances have been simulated to test the effectiveness of the proposed control method.

Based on the voyage data, the swaying motion of the UUV is simple harmonic motion with oscillation, the statistical maximum amplitude is 0.1396 rad and the period is 0.7 rad/s. Therefore, a function is introduced to represent $\theta_{ib}^{b}$.

(44) $$\left\{\begin{array}{l} {\theta_{ib}^{b}=\left(0.1134\sin\left(0.7t\right)+0.0262\mathrm{rand}\left(t\right)\right)\cdot \left[\begin{array}{ccc} 1 & 1 & 1 \end{array}\right]}^{T}\\ \omega_{ib}^{b}={\dot{\theta }}_{ib}^{b} \end{array}\right.$$

The friction torque, a nonlinear function with a certain bound, $T_{df}$ is mainly composed of the angular contact bearing and mass imbalance, $T_{da}$ is mainly composed of the angular contact bearing and mechanical seal device, and they are expressed as

(45) $$T_{df}=0.04\mathrm{rand}\left(t\right)-0.5+0.1\cos\beta$$

(46) $$T_{da}=1.0\left(\mathrm{rand}\left(t\right)-0.5\right)+12.4\left(\mathrm{rand}\left(t\right)-0.5\right)$$

The armature friction torque of the two DC motors is constantly changing with its rotation speed, therefore, a cosine function is proposed to represent $T_{dmi}$, that is,

(47) $$T_{dmi}=\max(T_{dmi})\cos\left(\omega_{i}t\right)\;\;\;\left(i=f,a\right)$$

The wave torque acting on the azimuth gimbal is periodic, and its maximum value under the level 4 sea conditions is calculated by the Morrison formula [33]. Therefore, a sine function is proposed to represent $T_{w}$, that is,

(48) $$T_{w}=0.5\sin\left(0.6t\right)+0.05\left(\mathrm{rand}\left(t\right)-0.5\right)$$

.

The $\Delta F_{i}$ and $\Delta D_{i}$ are mainly caused by the unknown inertial products of the two gimbals and system parameters variation, and they are related to the relative angular displacements of gimbals. Based on the derivation of the previous dynamic model, in simulation, they are represented as

(49) $$\Delta F_{i}=F_{i}\left(\sin2\alpha +\sin\alpha +\cos2\beta +\cos\beta \right)\left(i=1,2\right)$$

(50) $$\Delta D_{i}=D_{i}\left(\sin2\alpha +\sin\alpha +\cos2\beta +\cos\beta \right)\left(i=1,2\right)$$

The adaptive RBFNN contains 100 nodes, which are evenly spaced throughout the possible space of pitch and azimuth. The width of the RBFNN is set as 1.0, it can provide sufficient overlap between the radial basis functions. The initial weight matrix of the adaptive RBFNN is set as zero, and the weight matrix can be updated online by the approximation law in Equation (41). Then the adaptive RBFNN generates the corresponding estimated nonlinear term and disturbance term. Combining the adaptive RBFNN and the SMC, the optronic mast can generate the corresponding control commands for the motors to stabilize the LOS.

**Figure 4:**
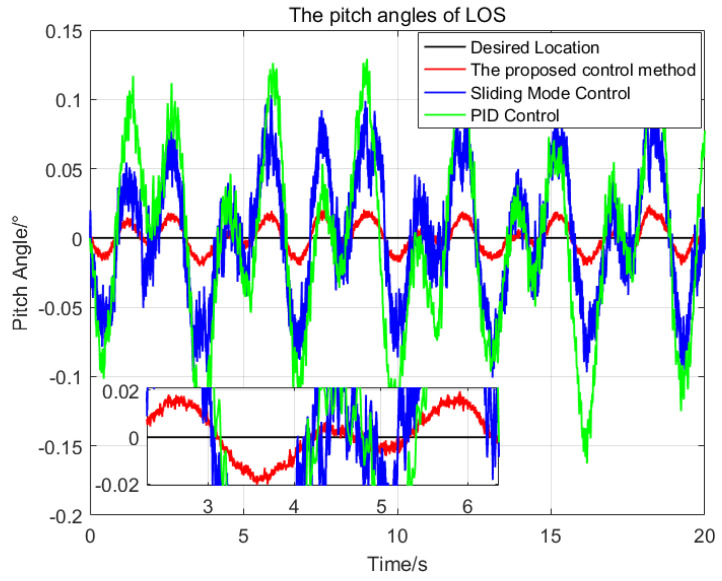
The comparison of the pitch angles of the LOS generated by the three control methods.

**Figure 5:**
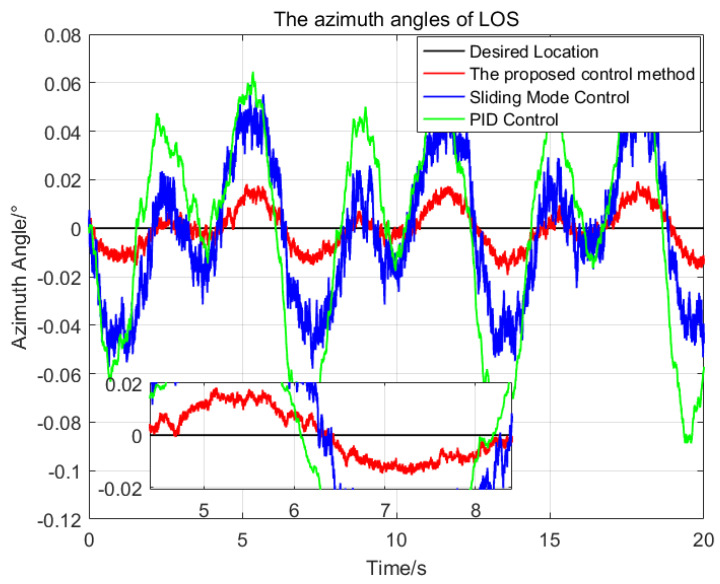
The comparison of the azimuth angles of the LOS generated by the three control methods.

To illustrate the performance of the proposed control method, a series of comparisons of the proposed control method, the sliding mode control method and the PID control method have been done. The same parameters have been chosen for the SMC and the proposed control method, and the PID parameters are optimal. At first, the desired attitude angles and the initial attitude angles of the LOS are all set as zeros, then the attitude angles of the LOS resulting from the application of the three control methods are shown in Figure 4 and Figure 5, respectively.

In Figure 4 and Figure 5, the red solid line, blue solid line and green solid line represent the trajectories of the LOS generated by the proposed control method, sliding mode control method and PID control method, respectively. The statistical results of the three control methods are shown in Table 2. For the pitch angles of the LOS, the maximum deviation from the desired pitch angle of the proposed control method is $0.024^{\circ }$, which is 23.3% of the SMC and 14.7% of the PID respectively. The standard deviation of the proposed control method is $0.01^{\circ }$, which is 22.2% of the SMC and 14.9% of the PID, respectively. For the azimuth angle of the LOS, the proposed control method has the best control performance, with the maximum deviation of $0.019^{\circ }$ and the standard deviation of $0.008^{\circ }$ from the desired azimuth angle.

Due to the nonlinearity and parameters variation of the system, it is difficult to choose a set of suitable control parameters for the PID control method, the maximum deviation from the desired attitude angles of the pitch and azimuth of the LOS are $0.163^{\circ }$ and $0.1^{\circ }$ respectively, which seriously affect the imaging quality of the imaging payloads. The SMC is insensitive to the parameters variation and composite disturbance, so its control performance is better than that of the PID control method.

Compared with the SMC, the proposed control method using adaptive RBFNN can approximate the ideal model of the nonlinearity and coupling disturbance of the system online, and avoid the large switching gains of the system. In addition, the proposed control method reduces the chattering phenomenon further by using a saturation function replace the sign function. Therefore, the proposed control method has better control performance than the SMC.

**Figure 6:**
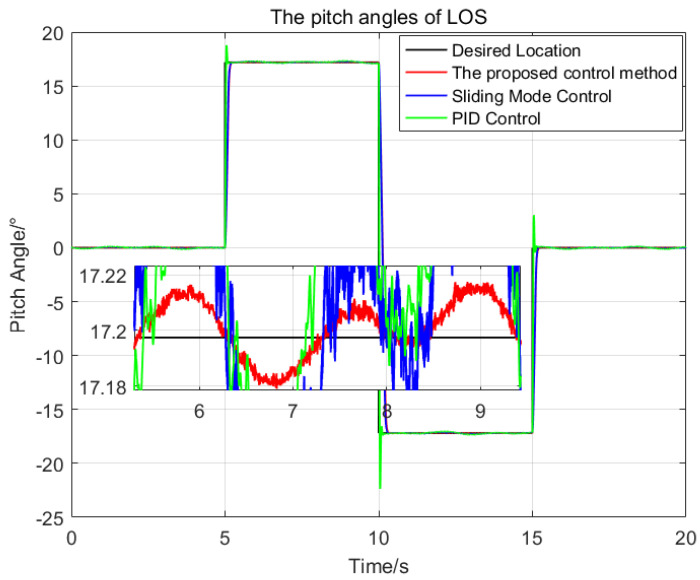
The comparison of the pitch angles of the LOS generated by the three control methods.

**Figure 7:**
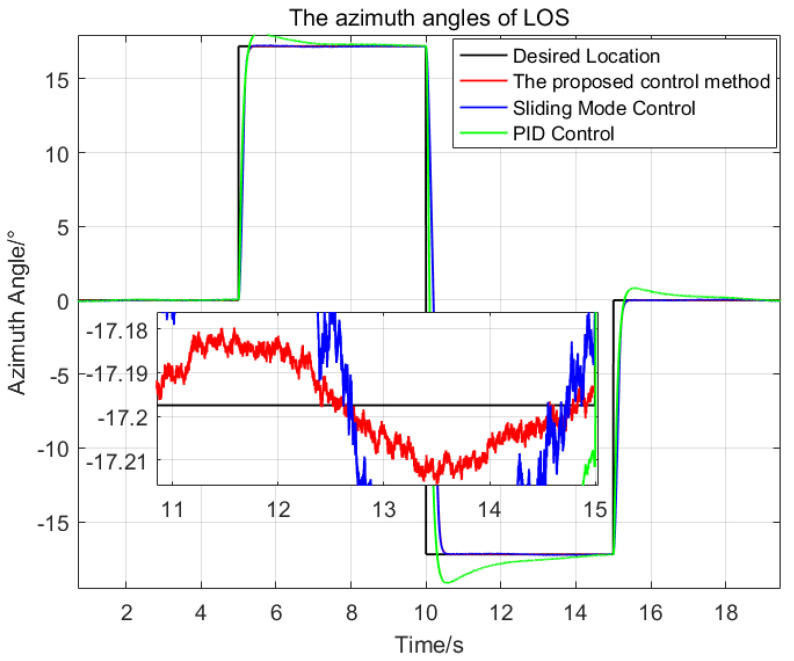
The comparison of the azimuth angles of the LOS generated by the three control methods.

Then, the desired LOS attitude angles of the optronic mast are set as a series of angular motion curves, and the initial values are all set as zeros. The comparison of the three control methods is shown in Figure 6 and Figure 7.

The settling time of the proposed control method is about 0.6 s. The maximum deviation from the desired pitch angles is $0.021^{\circ }$, which is 21.2% of the SMC and 0.4% of the PID respectively; The maximum deviation from the desired azimuth angle is $0.019^{\circ }$, which is 32% of the SMC and 1.0% of the PID, respectively.

Therefore, the proposed control method has a good dynamic response speed, and better stabilization precision than the two other control methods. The proposed control method meets the demands of the real application under high oceanic conditions and large swaying of the platform.

### 4.3. Experiments

**Figure 8:**
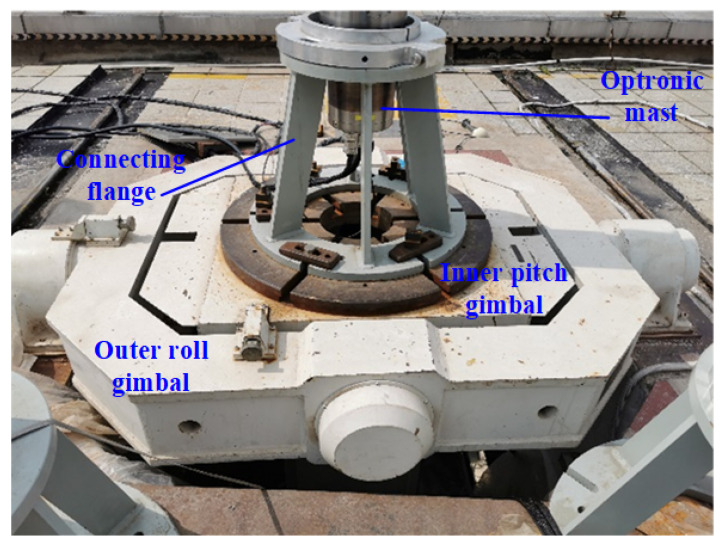
The swaying test platform experiment system.

The effectiveness of the proposed control method was tested by the swaying test platform. The swaying test platform installed on the roof with a good view consists of an outer roll gimbal and an inner pitch gimbal, which can simulate the swaying motion of the UUV. The maximum attitude error of the swaying test platform is $50^{\prime \prime }$, the attitude data update frequency is 100 Hz, and the maximum swaying amplitude is $10^{\circ }$. The optronic mast was installed on the inner gimbal of the swaying test platform by a flange to test, as shown in Figure 8.

**Figure 9:**
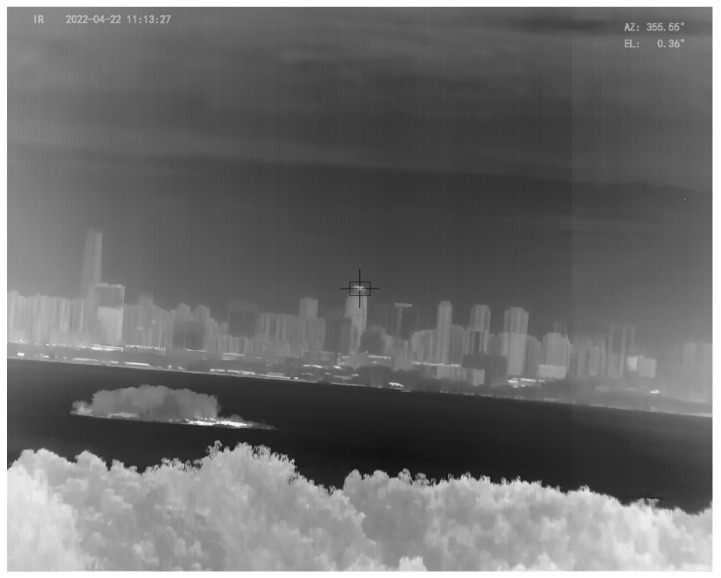
The image rotation phenomenon in the experiment.

The amplitudes of pitch and roll of the swaying test platform were all set as $8^{\circ }$, the swaying period was set as 9 s, and the phase difference of pitch and roll was randomly selected. Before the swaying experiment, the azimuth coordinates of the optronic mast and the swaying test platform were aligned by a reference target on the earth. Then the LOS of the optronic mast was adjusted to aim at a target with azimuth angle $355.53^{\circ }$ and pitch angle $0.32^{\circ }$ on the earth, and then the swaying test platform experiment was started. The attitude angles of the swaying test platform represent the swaying angles of the UUV, and is sent to the optronic mast in real time. With the acquired attitude angles, the proposed control method is used to accurately control the servo motors of the azimuth axis and the pitch axis of the optronic mast, so as to isolate the swaying disturbance coupled to the LOS of the optronic mast and keep the LOS steadily pointing at the selected target. Since the optronic mast is a two-axis ISP, it can only realize disturbance isolation in the azimuth and pitch directions, but not in the roll direction, which can cause the image rotation, as shown in Figure 9.

**Figure 10:**
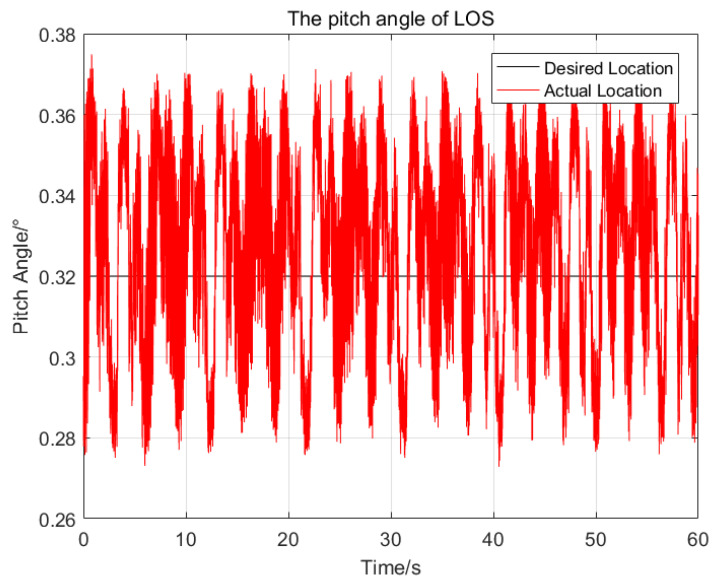
The experimental result of the pitch angle generated by the proposed method.

**Figure 11:**
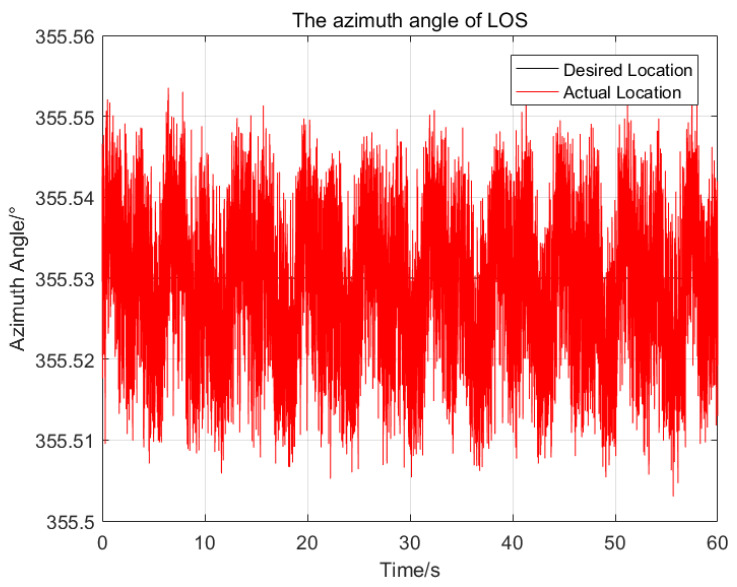
The experimental result of the azimuth angle generated by the proposed method.

In the infrared image shown in Figure 9, the cross-wire is the image center and represents the LOS; the black box is the target wave gate, and the gate center is the target direction. The instantaneous field of view (IFOV) of the infrared camera corresponding to the infrared image is $0.01^{\circ }$. In the experiment, the target miss distance (i.e., the number of pixels of the gate center deviating from the cross-wire center) is extracted in real time, and then converted into the attitude angle errors of the LOS with the IFOV, so as to obtain the movement trajectory of the LOS in pitch and azimuth, as shown in Figure 10 and Figure 11.

The maximum deviations from the desired attitude angles of the pitch and azimuth are $0.06^{\circ }$ and $0.027^{\circ }$, respectively; the standard deviations of the pitch and azimuth are $0.025^{\circ }$ and $0.0085^{\circ }$, respectively. The experimental results illustrate the effectiveness of the proposed control method for the LOS stable control under a big swaying movement, and the stabilization precision exceeds the actual demands.

**Figure 12:**
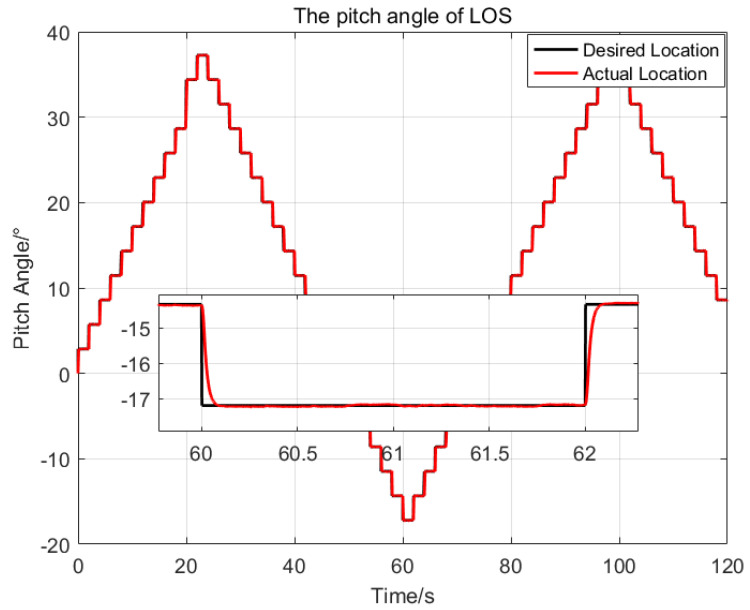
The tracking trajectory of the pitch with the proposed method.

**Figure 13:**
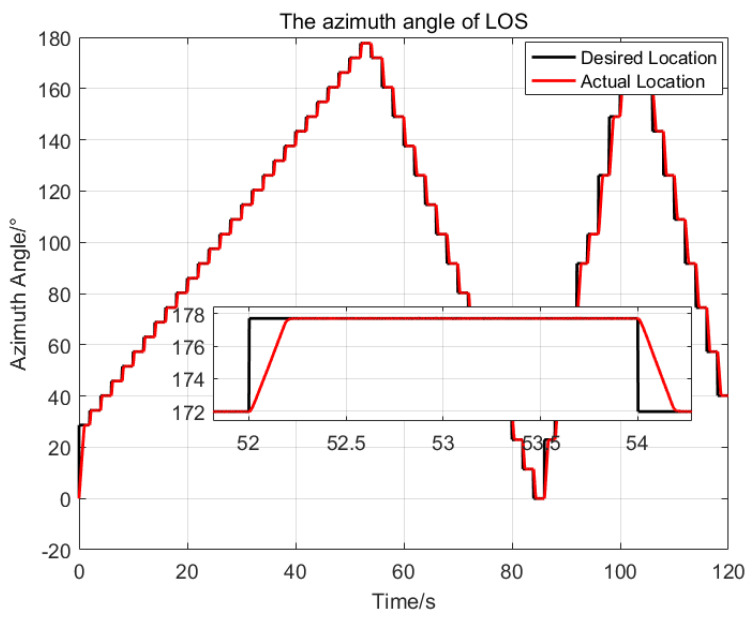
The tracking trajectory of the azimuth with the proposed method.

Then, an injection target tracking experiment was introduced, to test the dynamic response characteristic of the proposed control method. First, a target angle trajectory set was preset according to the pitch range ${\left[\begin{array}{cc} -20 & 40 \end{array}\right]}^{\circ }$ and azimuth range ${\left[\begin{array}{cc} 0 & 360 \end{array}\right]}^{\circ }$ of the LOS for the optronic mast. Then, the target with pixels of 6×6 is marked along the preset trajectory on the blank images with a frame rate of 50Hz. Finally, the target video was injected into the optronic mast system, and the swaying test platform was started. At this time, the LOS of the optronic mast is stable under the swaying movement from the previous test. The image processing unit of the optronic mast continuously captures the injected target and outputs the miss distance of the target in real time. Taking the output miss distance as the target attitude angle, the LOS catches up with the target attitude angle in real time under the proposed control method, so as to achieve target tracking. The tracking results obtained are shown in Figure 12 and Figure 13.

During the tracking experiment, the optronic mast realized the fast dynamic response and high stabilization precision control performance by the proposed method. The setting time of the system is about 0.3 s, and the standard deviations of pitch and azimuth are $0.036^{\circ }$ and $0.045^{\circ }$ respectively.

## 5. Conclusions

In this paper, a composite control method based on adaptive RBFNN and SMC was proposed. In this control method, the ideal model of a nonlinear and parameters-varying system is locally approximated by the adaptive RBFNN, so that the chattering amplitude is reduced in SMC. In addition, a saturation function instead of the sign-switching function is used to further reduce the chattering phenomenon in SMC. In the LOS stabilization experiment of the swaying test platform, the maximum deviation and the standard deviation from the desired attitude are $0.065^{\circ }$ and $0.02^{\circ }$, respectively. In the target tracking experiment of the swaying test platform, the maximum response speed of the LOS is 31.8∘/s, the standard deviations of the pitch and azimuth are $0.036^{\circ }$ and $0.045^{\circ }$ respectively, which realize the fast dynamic response and high stabilization precision control performance of the optronic mast. Compared with the previous methods used for the LOS stabilization control of optronic mast, the proposed control method has stronger anti-disturbance, stability and accuracy, and is feasible in engineering.

Although the proposed composite control method has achieved good effects on an example of a optronic mast, the good stable time of the system is achieved by debugging parameters rather than by strict design, so there may be some limitations in the application of this method. Based on this, how to design the composite control in finite time will be a future research direction.

## Figures and Tables

**Table 1:** The electrical parameters of the two DC torque motors.

Parameter	Pitch Motor	Azimuth Motor	Unit
$k_{t}$	1.3	1.4	Nm/Amp
$k_{e}$	0.017	0.13	Vs/rad
$R_{m}$	8.41	1.7	Ohms
$J_{m}$	$4.14\times 10^{-5}$	0.01	Kgm^2^

**Table 2:** The comparison of stabilization control performance of the three control methods.

Items	Pitch Axis		Azimuth Axis	
	Max Deviation/°	Standard Deviation/°	Max Deviation/°	Standard Deviation/°
Proposed control method	0.024	0.010	0.019	0.008
Sliding mode control method	0.103	0.045	0.058	0.029
PID control method	0.163	0.067	0.100	0.044

## Data Availability

Not applicable.

## Abbreviations

The following abbreviations are used in this manuscript:

LOS	Line of sight
RBFNN	Radial basis function neural network
SMC	Sliding mode control
UUV	Underwater unmanned vehicle
ISP	Inertially stabilized platform
IMU	Inertial Measurement Unit
CESO	Cascade Extended State Observe
RCESO	Reduced-order Cascade Extended State Observe
AC	Adaptive control
